# Combination Therapy of Losartan and Fisetin Reduces Senescence and Enhances Osteogenesis in Human Bone Marrow–Derived Mesenchymal Stem Cells

**DOI:** 10.1155/term/9187855

**Published:** 2025-03-19

**Authors:** Haruki Nishimura, Yoichi Murata, Michael T. Mullen, Kohei Yamaura, Jacob Singer, Charles Huard, Dane R. G. Lind, William S. Hambright, Chelsea S. Bahney, Marc J. Philippon, Johnny Huard

**Affiliations:** ^1^Center for Regenerative and Personalized Medicine, Steadman Philippon Research Institute, Vail, Colorado, USA; ^2^Department of Orthopaedic Surgery, Orthopaedic Trauma Institute, University of California San Francisco (UCSF), San Francisco, California, USA; ^3^The Steadman Clinic, Vail, Colorado, USA

**Keywords:** bone marrow-derived mesenchymal stem cell, cellular senescence, fisetin, losartan, osteogenesis

## Abstract

Bone marrow–derived mesenchymal stem cells (BM-MSCs) are well established for their osteogenic potential but are prone to senescence with aging or in vitro expansion. Drug treatments that reduce cellular senescence may enhance the regenerative capacity of BM-MSCs. This study investigates the effects of losartan and fisetin, both separately and in combination, on cellular senescence and osteogenesis. Human BM-MSCs were exposed to low and high concentrations of each drug for 24 h. Our findings showed that high-dose losartan exhibited cytotoxicity, focusing subsequent analyses on the low doses. Both low-dose losartan and fisetin effectively mitigated cellular senescence, with combined treatment showing synergistic effects in reducing senescence markers. From these initial findings, subsequent experiments utilized low doses of both compounds to evaluate their effect on differentiation capacity. Our multimodal approach, incorporating flow cytometry, senescence-associated heterochromatin foci (SAHF) immunohistochemistry, senescence-associated secretory phenotype (SASP) quantification, and differentiation potential assays, revealed that the combination of 23.6 μM of losartan and 50 μM of fisetin was optimal for reducing cellular senescence and enhancing osteogenesis in BM-MSCs. These results support potential therapeutic strategies to counteract age-related declines in bone health and improve healing. By targeting cellular senescence while promoting osteogenesis, losartan and fisetin offer promising avenues for future research aimed at enhancing the regenerative capacity of BM-MSCs in the context of musculoskeletal regenerative medicine.

**Summary**

• This study addresses a critical challenge in regenerative medicine by investigating the combined benefit of losartan and fisetin on bone marrow-derived mesenchymal stem cells (BM-MSCs). The synergy of these compounds, identified at specific concentrations (23.6 μM losartan and 50 μM fisetin), not only reduces cellular senescence but also enhances the osteogenic potential of BM-MSCs. This dual-action approach presents a promising strategy to rejuvenate BM-MSCs for potential applications in bone health and fracture healing. The findings contribute valuable insights into pharmacological interventions, advancing our understanding of regenerative strategies for musculoskeletal health.

## 1. Introduction

Bone marrow–derived mesenchymal stem cells (BM-MSCs) are a promising cell source for regenerative medicine and tissue engineering applications. These multipotent cells have the ability to differentiate into various cell lineages, including osteoblasts and adipocytes [1]. Understanding the mechanisms that regulate the balance between osteogenesis and adipogenesis in BM-MSCs is crucial for developing effective strategies to promote bone regeneration and combat bone-related disorders. This is especially important in the setting of age-related bone diseases where there is a reduction in cellular osteogenic capacity, leading to decreased bone mineral density in osteopenia/osteoporosis, as well as an accumulation of fatty tissue within the bone marrow [2, 3]. This fatty infiltration might be related to a preferential switch of MSCs from osteogenesis toward adipogenesis [4–6]. Given the rapidly aging global population and increased global fracture burden, research that improves osteogenesis in aging could significantly improve the potential of regenerative therapies in the elderly [7, 8].

In this study, we aim to test the efficacy of two clinically available drugs in the context of aged bone regeneration. Specifically, we explore drugs known to activate the osteogenic pathway and reduce inflammation by selectively eliminating senescent cells. Cellular senescence is defined as the age-related pathological accumulation of cells that cease dividing, resist apoptosis, and undergo significant alternations in gene expression such that their secretome is defined by a predominantly proinflammatory phenotype that is known to negatively impact bone health and bone healing [8, 9]. Senolytics and senomorphics are a class of drugs that aim to selectively eliminate senescent cells by targeting different pathways in the development of cellular senescence as recently detailed [10, 11]. This study explores the novel combination of drugs that may both eliminate senescent cells and reduce inflammation to promote bone regeneration.

The first drug we investigate is losartan, a selective angiotensin II receptor blocker that is FDA-approved for hypertension treatment and has shown promising effects in reducing fibrosis, promoting, and eliminating senescence [12–14]. Secondly, we test fisetin, a naturally occurring flavonoid found in various fruits and vegetables that has gained attention as a possible senolytic and for its regenerative capacity. Studies have demonstrated that fisetin exhibits antiaging, antioxidant, anti-inflammatory, and anti-cancer activities [15–17]. Additionally, fisetin has been shown to promote osteogenic differentiation and mineralization of BM-MSCs while inhibiting adipogenesis, suggesting its potential as a pro-osteogenic agent [18–20].

Given the individual effects of losartan and fisetin on osteogenesis and cellular senescence, we aimed to investigate their combined efficacy and potential synergistic effects on BM-MSCs. We hypothesized that the combination of fisetin and losartan could reduce cellular senescence in BM-MSCs and enhance osteogenesis while suppressing adipogenesis. To test this hypothesis, we first perform a dose–response study to define any potential cellular toxicity of the drugs alone or in combination. Then, we define changes to cellular senescence and osteogenic differentiation in vitro with this novel combination therapy. Our results indicate that both drugs have a beneficial effect of reducing cellular senescence and improving the osteogenic potential of BM-MSCs. This study supports that the combination of these drugs may enhance bone regeneration and serve as an innovative treatment method to address age-related changes in bone health.

## 2. Materials and Methods

This study conformed to the US Federal Policy for the Protection of Human Subjects.

### 2.1. Cell Culture and Expansion

Banked human BM-MSCs were purchased from ATCC (ATCC, catalog # PCS-500-012) and cultured in growth media containing DMEM-low glucose (Gibco, catalog # 11885084) with 10% fetal bovine serum (FBS; Gibco, catalog # 10437028) and 1% penicillin/streptomycin (P/S; Gibco, catalog # 15140148). Donor ages ranged from 19 to 35 years, with three females and one male donor. BM-MSCs from either three or four donors were used for each experiment. All cells were maintained in tissue culture-treated T75 flasks (Corning, catalog # 10-126-11) at 37°C in humidified air containing 5% CO_2_. All cells were received at passage 1 and subsequently passaged up to 4 times. At 70%–90% confluency, the cells were passaged and re-seeded at 250,000 cells per T75 flask. Cells on passage 5 were used for experimental treatment.

### 2.2. Losartan and Fisetin Treatments

**Figure 1:**
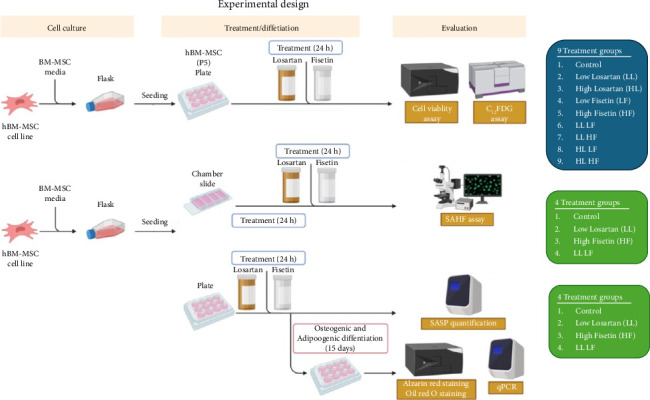
Experimental design. Experimental overview of this study. Human bone marrow-derived mesenchymal stem cells (BM-MSCs) on Passage 5 were used for all experiments (*N* = 4 donors). BM-MCSs were plated in a 12-well plate and first accessed for viability assay and senescence by quantifying C_12_FDG using flow cytometry assays under nine different treatment conditions (including control; see treatment group details in the blue inset). Subsequently, more detailed senescence phenotyping (senescence-associated heterochromatin foci (SAHF) assay and senescence-associated secretory phenotype (SASP) quantification) and differentiation were completed on a subset of four treatment groups (including control; see treatment group details in the green inset). Figure created in BioRender.com.

Cells on passage 5 were seeded into 12-well plates (Genesee, catalog # 25106) at a density of 40,000 cells/well, chamber slides (ThermoFisher, catalog # 177399) as well at a density of 5000 cells/cm^2^ only for senescence-associated heterochromatin foci (SAHF) assay, in triplicate for each group. After the cells adhered to the plate or the chamber slide, the media was changed. For the control group, normal BM-MSC growth media was used (DMEM-low glucose with 10% FBS and 1% penicillin/streptomycin). For the treatment groups, the same control media was used, but either *Losartan* (USP, catalog # 1370462) or *Fisetin* (Selleckchem, catalog #S2298) was added to the media for 24 h. Losartan was tested at low (23.6 μM low losartan, “LL”) and high (236.5 μM, high losartan, “HL”) doses. Similarly, fisetin was tested at a low (50 μM, low fisetin, “LF”) or high (100 μM, high fisetin, “HF”) dose. The combination of these drugs included LLLF, LLHF, HLLF, and HLHF, as detailed in Figure 1. Cells were treated once for 24 h, after which the wells were washed, and control media was added.

### 2.3. Cell Viability Assay

Cell viability was quantified using the PrestoBlue Cell Viability Reagent (Invitrogen, catalog # A13262) for losartan and fisetin at nine different concentration combinations. 1000 μM of fisetin was also tested for toxicity. The cells were incubated in the PrestoBlue reagent for 2 h before stopping the reaction and quantifying metabolic activity. 200 μL of media was transferred to a 96-well plate (Greiner, catalog # 650001) to read the optical density at 570 nm as per the manufacturer's protocol using the Tecan Infinite 200 Pro M Plex Microplate Reader (Tecan, Männedorf Switzerland). Cells were imaged using a Nikon Eclipse Ti brightfield microscope, photographed with an attached Nikon DS-Ri2 camera. Contrast and brightness of the JPEG images were edited in PowerPoint to improve the visualization/sharpness of the cells.

### 2.4. C_12_FDG Quantification

**Figure 2:**
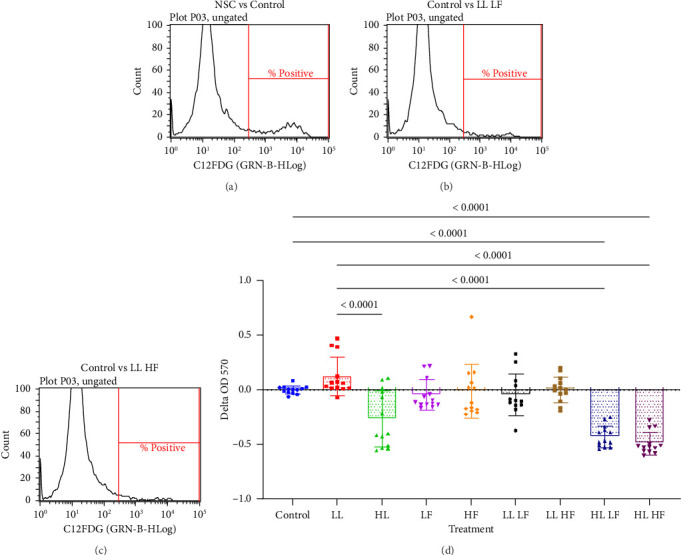
C_12_FDG flow cytometry of BMSCs treated with losartan and fisetin. (a)–(c) Representative histograms representing the gating strategy. (a) Histograms for non–stained cells (NSCs, gray) were overlaid with histograms for untreated control cells (control, light red) to set the threshold for C_12_FDG positive cells. (b, c) Representative histograms using this threshold comparing cells treated with low losartan and low or high fisetin (LL LF/HF, green, Figure (b) and (c), respectively) to untreated control cells (control, light red). (d) Delta percent positive for C12FDG after losartan and fisetin treatment. Five thousand events were recorded per sample, with three samples being used for each group. Data are presented as the mean ± standard deviation. Significant difference from control ⁣^∗∗∗^*p* < 0.001 and ⁣^∗∗∗∗^*p* < 0.0001. LL: low losartan (23.6 μM); HL: high losartan (236.5 μM),; LF: low fisetin (50 μM); HF: high fisetin (100 μM).

Cells positive for senescence-associated β-galactosidase were quantified with C_12_FDG using flow cytometry as previously reported [21–24]. C_12_FDG is a compound that fluoresces at 514 nm when hydrolyzed by intracellular senescence-associated β-galactosidase; elevated senescence-associated β-galactosidase activity is a characteristic of senescent cells. To quantify C_12_FDG, BM-MSCs plated in 12-well plates after losartan and fisetin treatments were treated with 100 nM of bafilomycin A1 (Cell Signaling, catalog # 54645) for 1 h at 37°C and 5% CO_2_. Next, 33 μM C_12_FDG (Thermo Fisher, catalog #D2893) was added and incubated for 1 h. The cells were then washed with PBS and collected from the plate using the TrypLE reagent (Gibco, catalog # 12605028). Using unstained cells to set gate thresholds (Figure 2(a)), we quantified C_12_FDG positive cells with a Guava EasyCyte flow cytometer (Luminex, Austin, TX). Each donor BMSC was measured in triplicate, with 5000 events being recorded per replicate.

### 2.5. SAHF Staining

Cells were plated in triplicate into chamber slides for SAHF staining. The following day, cells were randomly assigned into LL, LF, and LLLF treatment groups and treated with losartan and/or fisetin for 24 h. Following treatment, the cells were washed twice with 1X PBS, fixed with cold 4% paraformaldehyde (Alfa Aesar, Catalog #J19943K2) for 15 min, and then washed three more times with PBS. Cells were blocked using 10% donkey serum (DS; Jackson Immuno, catalog # 017000121) and then permeabilized with 0.3% Triton X-100 (Fisher, catalog # BP151-500) in 1X PBS for 1 h before overnight incubation at 4°C with the γ-H2AX antibody (Millipore Sigma, catalog # 05636I) and the H3K9me3 antibody (Millipore Sigma, catalog # 07442) using a 1:150 dilution and a 1:250 dilution, respectively. Both antibodies were added in dilution buffer containing 1X PBS with 1% DS, 1% bovine serum albumin (BSA; Sigma, catalog # A9647-100G), and 0.3% Triton X-100. Following antibody conjugation, the cells were washed three times with wash buffer containing 1X PBS with 0.1% BSA. The cells were then incubated with 1:400 diluted AlexaFlour 488 and 594 (Invitrogen, catalog # A21206 and A21203, respectively) secondary antibodies for 1 h at room temperature. The cells were washed three more times and incubated with 1 μg/mL of DAPI (Sigma, catalog #D9542-10 MG) for 10 min before being washed with PBS. Five images were taken per well, and three wells per group were imaged using a Nikon Eclipse Ni-U microscope. Cells positive for the γ-H2AX and H3K9me3 antibodies were manually counted using the Cell Counter in ImageJ software by two blinded observers (H.N. and C.H.).

### 2.6. Senescence Phenotype

Cells were grown to confluency using normal growth media in 12-well plates. Once all wells reached a 70%–90% confluency, the cells were treated with low losartan (LL) and/or low fisetin (LF) for 24 h. Quantification of canonical SASP genes *Interleukin-1β (IL-1β), IL6*, and *transforming growth factor-β1* (*TGF-β1*) was performed by quantitative real-time polymerase chain reaction (qRT-PCR) following drug treatments. The detailed methodology for qPCR is described in the “RNA Isolation, Reverse Transcription, and Quantitative PCR” section.

### 2.7. Differentiation Assays

BM-MSC differentiation was carried out using the Lonza Osteogenic and Adipogenic kits (Lonza, catalog # PT3002 and PT3004, respectively) according to the manufacturer's instructions. Cells were grown to a 70%–90% confluency using a normal BM-MSC growth media (DMEM-low glucose with 10% FBS and 1% penicillin/streptomycin). Cells were then treated with low losartan and/or low fisetin for 24 h. On the following day, cells were washed twice and the media was changed to differentiation media. For osteogenic differentiation, fresh Lonza osteogenic induction media was added every 3 days for a total of 15 days. For adipogenic differentiation, the Lonza adipogenic induction media was added for 3 days followed by 2 days with the Lonza adipogenic maintenance media. This process was repeated for a total of 15 days. Alizarin Red staining and qPCR for collagen type I alpha (COL1A), osteocalcin (OC), and alkaline phosphatase (ALP) were performed after osteogenic differentiation as detailed below. Further, Oil Red O staining and qPCR for *fatty acid binding protein (FABP) 4, peroxisome proliferator-activated receptor γ* (PPARγ), and *CCAAT/enhancer binding protein (CEBP*) were performed after adipogenic differentiation.

### 2.8. Alizarin Red Staining

Following osteogenic differentiation, the cells were fixed with cold 4% paraformaldehyde (Alfa Aeser, Catalog #J19943K2) for 15 min and washed twice with water. The cells were then incubated at room temperature for 30 min with a 2% Alizarin Red (Sigma-Aldrich, catalog # A5533) solution in water with pH adjusted to 4.1–4.3 using ammonium hydroxide. The cells were then washed 5 times with water and imaged using a Nikon Eclipse Ni-U microscope. To quantify the Alizarin Red staining, the dye was extracted by incubating the cells for 30 min at room temperature with 10% acetic acid while shaking. The cells and acetic acid solution were then collected into microcentrifuge tubes using a cell scraper. The samples were then vortexed and heated at 85°C for 10 min before being cooled on ice for 5 min. Next, the samples were centrifuged at 20,000 × g for 15 min. Following centrifugation, the supernatant was collected and brought to a pH of 4.1–4.5 using 10% ammonium hydroxide. The final solution from each sample (200 μL) was then added to a 96-well plate (Greiner), and the optical density was measured at 405 nm using a Tecan Infinite 200 Pro M Plex Microplate Reader (Tecan, Männedorf Switzerland).

### 2.9. Oil Red O Staining

Following adipogenic differentiation, the cells were fixed with cold 4% paraformaldehyde (Alfa Aeser, Catalog #J19943K2) for 15 min and washed twice with water. The cells were then incubated with 60% isopropanol for 5 min. The cells were then incubated with 500 μL of Oil Red O working solution for 15 min at room temperature. The working solution was made from 3 parts of Oil Red O stock in 2 parts of water and filtered through Whatman No. 1 filter paper prior to use. Oil Red O stock was made from 60 mg of Oil Red O (Sigma-Aldrich, catalog # MAK194C) in 20 mL of 100% isopropanol. Following incubation, the cells were washed 3 times with distilled water. The cells were then imaged using a Nikon Eclipse Ni-U microscope. Following imaging, the dye was collected by adding 500 μL of isopropanol per well and incubating on a rocker for 15 min; 200 μL of dye from each well was then collected into a 96-well plate, and the optical density was measured at 492 nm using the Tecan Infinite 200 Pro M Plex Microplate Reader (Tecan, Männedorf Switzerland).

### 2.10. RNA Isolation, Reverse Transcription, and Quantitative PCR

Total RNA was extracted using the Trizol reagent (Invitrogen, catalog # 15596026) according to the manufacturer's instructions. Following RNA isolation, reverse transcription was performed using the qScript cDNA synthesis kit (VWR, catalog # 95048-100) according to the manufacturer's instructions. Quantitative real-time polymerase chain reaction (qRT-PCR) was performed using the PerfecCTa SYBR Green FastMix (VWR, catalog # 101414-280) on the Step-One Plus Real-Time PCR system (Applied Biosystems, catalog # 4376598). All results were normalized to GAPDH as the housekeeping gene, and the results were plotted as 2^−ΔCT^. Primer sequences are shown in Table 1.

### 2.11. Statistics

All statistical comparisons and figures were generated using the PRISM 9 analysis software (GraphPad software, CA, USA). Data in each graph are presented as the mean ± standard deviation with individual data points plotted. One-way analysis of variance (ANOVA) was completed to test for significant differences between the treatment groups, followed by Tukey's honestly significant difference (HSD) post hoc testing if indicated by ANOVA. Values with a *p* < 0.05 were considered statistically significant. Exact *p* values are included in the figures.

## 3. Results

### 3.1. High Doses of Losartan and Fisetin Have a Detrimental Effect on Cellular Function

**Figure 3:**
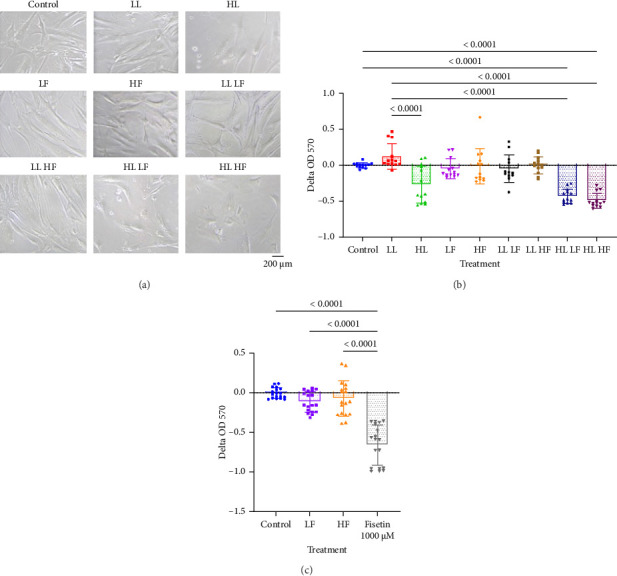
Cell viability assay of bone marrow–derived mesenchymal stem cells after losartan and fisetin treatment. (a) Representative morphological features of the cells. (b, c) PrestoBlue cell viability reagent assay for passage 5 cells with various concentrations of losartan and fisetin. LL: low losartan (23.6 μM); HL: high losartan (236.5 μM); LF: low fisetin (50 μM); HF: high fisetin (100 μM).

The first phase of this study investigated whether either losartan or fisetin had toxicity limits that negatively impacted cellular proliferation. Losartan was tested at a low (23.6 μM low losartan, “LL”) and high (236.5 μM, high losartan, “HL”) dose. Fisetin was tested at a low (50 μM, low fisetin, “LF”) and high (100 μM, high fisetin, “HF”) dose. Drug treatment was compared to a control condition in which the BM-MSCs received the base growth media without either losartan or fisetin. Images of representative cells following 24 h of each treatment are shown in Figure 3(a). Treatment with HL resulted in fewer cells compared to other treatment groups and presented with an apoptotic phenotype. PrestoBlue cell viability assay confirmed that treatment with HL significantly decreased the BM-MSC proliferation potential compared to control and LL groups (Figure 3(b)). As we did not see any negative impact from fisetin at 100 μm, we chose to test whether we could find a toxicity limit using a higher dose. We found that at 1000 μm, a 10-fold higher dose of fisetin, we did see a statistically significant reduction in cell number (Figure 3(c)).

### 3.2. Both Losartan and Fisetin Demonstrate the Ability to Reduce MultiModal Measures of Senescence

We next aimed to explore how losartan and fisetin, either individually or in combination, impacted cellular senescence. First, we measured cellular senescence with flow cytometry using the fluorescent-activated β-galactosidase compound C_12_FDG (Figures 2(a), 2(b), and 2(c)). All treatment groups, including fisetin, significantly reduced the percentage of C_12_FDG positive cells when compared to the control group (Figure 2(d)). The low versus high dose of fisetin showed similar effects on senescence. Losartan on its own did not significantly reduce the percent of C_12_FDG positive cells.

**Figure 4:**
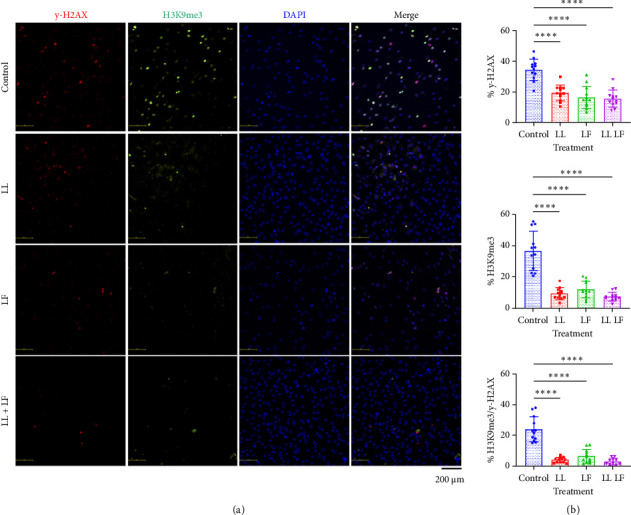
Senescence-associated heterochromatin foci assay of bone marrow–derived mesenchymal stem cells after losartan and fisetin treatment. (a) Representative microscopic images of g-H2AX, H3K9me3, DAPI, and merged. (b) Positive rate of g-H2AX, (c) 3K9me3, and (d) those double positive for both g-H2AX and 3K9me3. Data are presented as the mean ± standard deviation. LL: low losartan (23.6 μM); LF: low fisetin (50 μM).

We then used immunohistochemistry to visualize and semi-quantitate BM-MSCs positive for SAHF markers (Figure 4(a)). DNA damage and the formation of SAHF are proposed as causal mechanisms in the induction of cellular senescence [25, 26] and, therefore, serve as an alternative to C_12_FDG quantification for the identification of senescence. We specifically looked at two markers: γH2AX and H3K9me3. Phosphorylation of the serine on residue 139 of the histone variant H2AX, forming the γH2AX, is a common marker of cellular senescence and is a highly sensitive molecular marker of DNA damage and telomere shortening in response to the induction of double-stranded breaks [27, 28]. H3K9me3 is another marker associated with heterochromatin that tri-methylates histone H3, resulting in epigenetic modification of DNA packaging. In the control group, we saw that an average of 35 ± 7% cells was identified as positive for γH2AX (Figure 4(b)) and similarly 37 ± 13% cells were identified as positive for H3K9me3 (Figure 4(c)). There were less cells double-positive for both γH2AX and H3K9me3 cells with the average at 24 ± 8% across four different BM-MSC donors (Figure 4(d)). Importantly, we found that both the cells positive for either individual or both SAHF markers were significantly reduced following LL, LF, or LLLF treatment compared to the control group (Figure 4(d)). However, there was no significant difference in SAHF levels between any of the treatment groups, indicating that they all impacted cellular senescence similarly.

### 3.3. Losartan and Fisetin Therapy Reduced IL-1β Gene Expression From Culture-Expanded BM-MSCs

**Figure 5:**
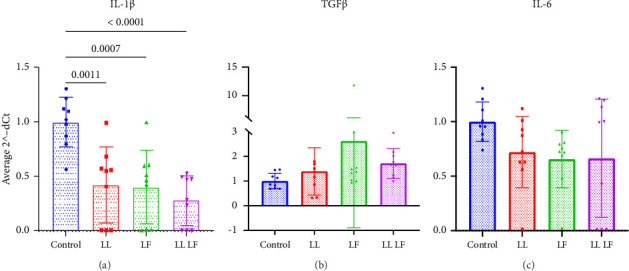
Gene expression of senescence-associated secretory phenotype in bone marrow–derived mesenchymal stem cells after losartan and fisetin treatment. (a) IL-1β, (b) IL6, and (c) TGF-β1 gene expressions following treatment. Data are presented as the mean ± standard deviation. LL: low losartan (23.6 μM); LF: low fisetin (50 μM).

Since cellular senescence is highly associated with an increase in inflammatory markers and the expression of TGF-β1, we chose to explore how losartan and fisetin treatment modulated the expression of these canonical genes (Figure 5). We found that treatment with LL, LF, and LLLF significantly decreased the expression of *Interleukin-1β* (*IL-1β)* when compared to the control group (Figure 5(a)). Similar to other measures of senescence, we did not find any significant difference within the treatment groups. We did not see statistically significant differences in *IL6* (Figure 5(c)) or *TGF-β* (Figure 5(c)) gene expression with the treatment groups compared to the untreated control groups, perhaps due to large variations in some treatment groups despite the large number of replicates (*N* > 8).

### 3.4. Losartan and Fisetin Enhance the Osteogenic Potential of BM-MSCs

**Figure 6:**
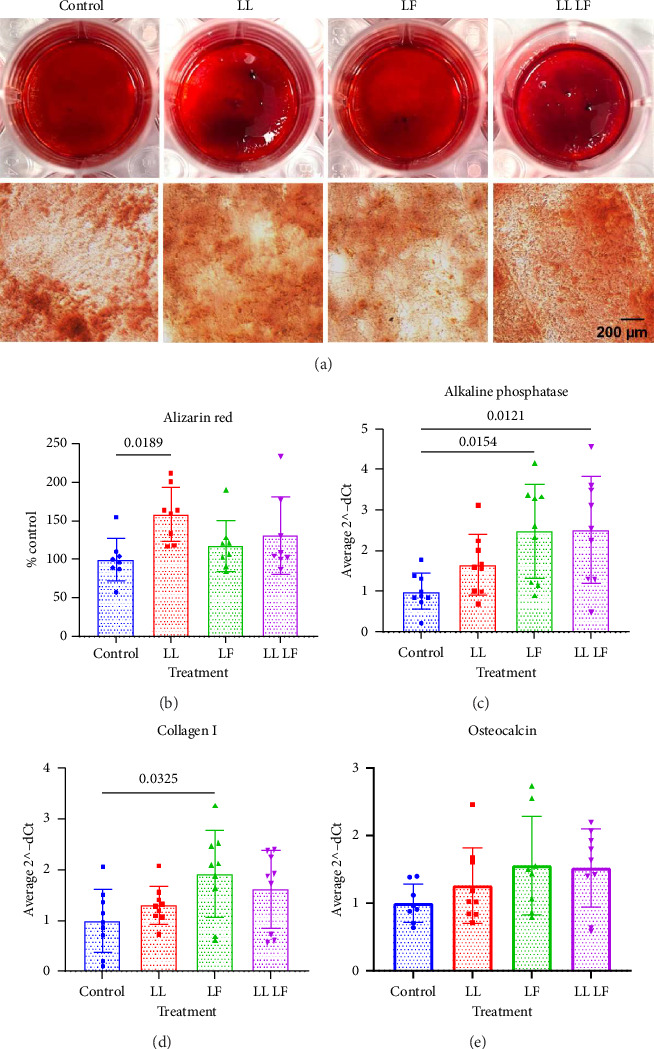
Osteogenic differentiation potential of bone marrow–derived mesenchymal stem cells after losartan and fisetin treatment. (a) Positive staining of colonies and representative microscopic images with Alizarin Red staining. (b) Quantitative Alizarin Red staining assay. (c)–(e) Gene expression of COL1A, OC, and ALP. From panels (b)–(e), data are presented as the mean ± standard deviation. LL: low losartan (23.6 μM); LF: low fisetin (50 μM).

We next aimed to understand if losartan and fisetin, either individually or in combination, changed osteogenic differentiation (Figure 6). The representative images of Alizarin Red staining (Figure 6(a)) show robust osteogenic differentiation in all treatment groups with a statistically significant increase in mineral deposition in the low losartan group following a quantitative Alizarin Red assay (Figure 6(B)). We also found that all treatments led to a statistically higher expression of the early osteogenic genes, *alkaline phosphatase* (*ALP*, Figure 6(c)) and *collagen type 1A* (*col1A1*, Figure 6(d)), but not the later osteogenic gene *osteocalcin* (*OC*, Figure 6(e)).

### 3.5. Losartan and Fisetin Did Not Impact the Adipogenic Differentiation of BM-MSCs

**Figure 7:**
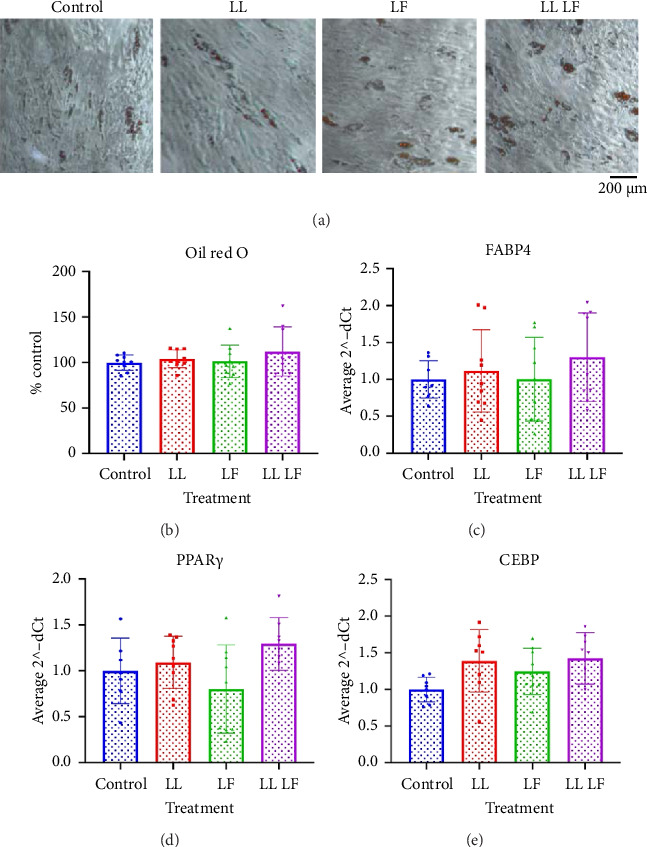
Adipogenic differentiation potential of bone marrow–derived mesenchymal stem cells after losartan and fisetin treatment. (a) Representative microscopic images of Oil Red O positive cells. (b) Quantitative Oil Red O staining assay. (c)–(e) Gene expression of FABP4, PPARγ, and CEBP. From panels (b)–(e), data are presented as the mean ± standard deviation. LL: low losartan (23.6 μM); LF: low fisetin (50 μM).

Lastly, we tested the influence of losartan and fisetin treatment on adipogenic differentiation (Figure 7). The representative images of Oil Red O staining for each treatment group (Figure 7(a)) and the quantitative Oil Red O assay (Figure 7(b)) did not show any significant difference among all groups. There was also no significant change in the gene expression of any of the adipogenic programs *fatty acid binding protein 4* (*FABP4*, Figure 7(c)), *peroxisome proliferator-activated receptor γ* (*PPARγ*, Figure 7(d)), and *CCAAT/enhancer binding protein*, (*CEBP*, Figure 7(e)), among all groups.

## 4. Discussion

The use of bone marrow-derived mesenchymal stromal or progenitor cells, commonly called BM-MSCs, has gained clinical interest in orthopedic applications due to their ability to promote tissue regeneration by reducing inflammation and multipotent differentiation into bone, cartilage, and fat cells. One limitation of this technology is obtaining sufficient numbers of BM-MSCs to promote tissue regeneration and concerns over heterogeneity across different patient donors. In vitro cellular expansion is frequently used to expand BM-MSCs for therapeutic applications, but this process is associated with significant changes in the cellular phenotype and secretome of the BM-MSCs [29]. One of the cellular changes commonly associated with in vitro cellular expansion is the accumulation of cellular senescence [29]. Aging is another risk factor for the accumulation of cellular senescence. In our published work, we have also found that chronological aging is associated with increased senescence in circulating blood cells, as measured by C_12_FDG [22]. However, this is not a perfect linear relationship, with some older donors behaving as younger and vice versa. While we presume this difference in blood is similarly transferred to BM-MSCs, comprehensive human studies are not available, and our ongoing work in large clinical cohorts is specifically investigating the correlation between chronological age and BM-MSCs. Cellular senescence and the production of senescence-associated secretory phenotype (SASP) may negatively affect the regenerative potential of BM-MSCs. In this study, we aimed to test whether either losartan or fisetin, or the combination treatment, could reduce the senescent phenotype of MSCs in vitro, with the goal of improving their regenerative capacity.

To optimize the dosage of these drugs, which have never been used in combination on BM-MSCs, we first explored different doses of losartan and fisetin on the function of BM-MSCs. Our results showed that treatment with 236.5 μM of losartan significantly reduced the cell viability of BM-MSCs, whereas 23.6 μM was not cytotoxic. While there are no previous reports studying the cell toxicity limit for BM-MSCs in vitro, Logan et al. reported that high-dose losartan at 100 mg/mL (236.5 mM) was similarly cytotoxic on in vitro-cultured human muscle-derived stem cells and that an intra-articular injection of 100-mg losartan significantly inhibited cartilage repair in a rabbit model [30]. Since the final concentration of losartan in the knee joint was not reported in their study, it is difficult to directly compare their in vivo data to our in vitro results, but together, these studies suggest that the toxicity of losartan needs to be monitored in a cell-specific manner. The cells tolerated fisetin better, with 100 μM and lower showing no cytotoxicity and a limit seen only when we tested a 10-fold higher dose.

After establishing the cytocompatibility limits, we next explored whether these doses of losartan and fisetin were effective in removing cellular senescence. Our results showed that both doses of fisetin, alone or in combination with losartan, significantly reduced cellular senescence as measured using C_12_FDG assay and SAHF staining. Losartan, on its own, was also effective at removing SAHF. Fisetin was first identified as a senolytic that extends the health and lifespan by eliminating aging cells in 2018 [17, 31]. Mechanistically, these studies found that fisetin significantly reduced the expression of p16^INK4A^ and downregulated the PI3K/AKT/mTOR and NF-κB pathways. While losartan is not typically viewed as a senolytic, the principal effector of the renin–angiotensin system is recognized to accelerate aging and cellular senescence [32]. Specifically, Zhou et al. demonstrated that angiotensin II treatment on human glomerular mesangial cells (GMCs) resulted in the accumulation of cellular senescence [14]. Feng et al. confirmed that angiotensin II was responsible for inducing telomere length shortening, P53 and P21 expressions, cell cycle arrest, and thus promotes cellular senescence in GMCs [33]. Furthermore, they revealed that losartan can delay aging by reducing the cellular senescence and suppressing telomere shortening by acting as an effective angiotensin II receptor antagonist [33].

In addition to reducing cellular senescence, losartan and fisetin lead to a significant reduction in the gene expression of IL-1β that was most effective when used as a combination therapy. This anti-inflammatory effect is supported by the literature, which has shown that losartan decreases the production of proinflammatory cytokines TNFα, IL-1β, and IL-6 [34, 35]. Silveira et al. demonstrated that treatment with losartan decreased the production of TNFα and IL-1β in antigen-induced arthritis mice [36]. Dionisio et al. also reported that pretreatment with losartan on rats with experimentally induced periodontitis led to the significant attenuation of IL-1β [37].

Fisetin, or 7,3′,4′-flavon-3-ol, is a naturally occurring polyhydroxy flavonoid that exhibits a multimodal anti-inflammatory and anti-oxidative activity, as recently reviewed [38–40]. Flavonoids act by downregulating the PI3 kinase, NF-κB, and RAS pathways to block the expression of the canonical proinflammatory pathways TNFα, IL-1β, IL-6, and inducible nitric oxide synthase (iNOS). Multiple other possible mechanisms for reducing inflammation and SASP are being explored with fisetin, but they have not been explored in a musculoskeletal or bone regeneration context.

Lastly, we aimed to ensure that these therapeutics did not negatively impact the osteogenic capacity of BM-MSCs. Our focus on bone was aimed at understanding the decline in bone health (osteoporosis) and diminished fracture healing that are linked to age. Specifically, decreased bone formation and increased adipogenic deposition are converse phenotypes commonly associated with aging [41, 42]. Interestingly, not only did treatment not decrease osteogenesis, but 23.6 μM of losartan enhanced osteogenic differentiation. Mechanistically, losartan appears to be working at multiple levels to improve osteogenesis. In vitro, 5 μm of losartan can increase osteoblast proliferation [43]. In vivo, losartan increased the expression of key osteogenic programs, including runt-related transcription factor-2 (RUNX2), alkaline phosphatase, and osteocalcin contributing to accelerated chondrocyte hypertrophy and increased mineral deposition [37, 44, 45]. Increased osteogenesis may be further enhanced by losartan's suppression of RANKL-induced ERK1/2 phosphorylation leading to reduced osteoclastogenesis and a synergistic improvement of bone mass [45]. Lastly, the inhibition of TGFβ by losartan has also been shown to block the high levels of TGFβ that are hypothesized to play a fundamental role in driving pathogenic mechanisms associated with bone disease osteogenesis imperfection and age-related impairment in fracture healing [43, 46].

Similarly, the positive effects of fisetin on bone health are also being explored and appear to promote osteoblast activity and inhibit osteoclasts. Molagoda et al. demonstrated that fisetin promotes the differentiation of the MC3T3-E1 preosteoblast cell line by stimulating the Wnt/β-catenin signaling through phosphorylation of glycogen synthase kinase 3 β at Ser9 [47]. Choi et al. reported that fisetin decreased osteoclast differentiation by downregulating NFATc1 signaling to attenuate the receptor activator of NF-κB [48, 49]. These in vitro studies support the in vivo effect of fisetin in preventing bone mineral density loss with aging [21].

Importantly, increased osteogenesis in our study was not associated with an increase in adipogenesis. While the effect of losartan and fisetin on adipogenic differentiation has not been well studied, Janke et al. have found that angiotensin receptor blockers could increase adipogenesis in human preadipocytes through the activation of PPARγ-target genes [50]. Their results showed that 10 μM, but not 1 μM, of losartan treatment enhanced adipogenesis. Our data did not find a significant increase in the PPARγ gene expression after low losartan treatment, suggesting that higher concentrations of losartan are required to enhance adipogenesis in BM-MSCs. Kim et al. showed that fisetin inhibited lipid accumulation and suppressed the expression of the 3T3-L1 fibroblast cell line often used to study cellular mechanisms associated with obesity [20]. They suggested that fisetin inhibits PPARγ transcriptional activity and suppresses adipogenesis by promoting Sirt1-mediated deacetylation of PPARγ and forkhead box O1 and by enhancing the association of Sirt1 with the PPARγ promoter. Although fisetin did not decrease adipogenesis in our study, its potential impact on the balance of osteogenesis to adipogenesis differentiation could be translated in vivo in aging studies administering these therapeutics.

Certain limitations were noted in the present study. One limitation is that here we only pre-treated BM-MSCs with losartan and fisetin before differentiation. As such, it is unclear what the effect of continuous treatments would be on trilineage differentiation. Further experiments would be needed to test how these drugs affect chondrogenic differentiation, although we have previously found a positive in vivo effect of losartan on cartilage repair [30, 51, 52]. We also only looked at the efficacy of our therapeutic treatments on in vitro expansion-induced cellular senescence and not other causes of cellular senescence such as chronological aging or disease. While we presume this treatment would work to reduce cellular senescence and increase homogeneity of the BM-MCS population regardless of the cause of senescence, further experiments are needed to support this premise. Specifically, demonstrating the efficacy of this combination treatment in chronologically aged cells or rodent models would build on our prior work showing that fisetin could attenuate age-related bone loss [21]. Additionally, our investigation into the anti-inflammatory potential of our combination therapy was limited to gene expression and a protein-level quantification of SASP.

## 5. Conclusion and Future Directions

The present study discovered a regenerative application of losartan and fisetin in combination for the improvement of osteogenesis in human BM-MSCs. Specifically, we found that lower doses of these drugs worked alone and synergistically to promote the osteogenic differentiation potential of BM-MSCs without increasing their adipogenic differentiation. Our data, together with the previous literature, suggest that these drugs work mechanistically to enhance bone formation by stimulating osteogenic gene expression to enhance mineralization and simultaneously inhibiting osteoclast activity.

We also found that losartan and fisetin combination therapy decreased cellular senescence and the expression of the inflammatory gene IL-1β. Future experiments to fully characterize the beneficial reduction in inflammation and SASP expression will further support this regenerative mechanism in the context of age-related bone repair. Furthermore, fisetin has also been shown to have anti-oxidative properties. While we did not look into reactive oxygen species (ROS) in this particular study, our previous study using adipose-derived mesenchymal stem cells (ADSCs) reported that 50 M of fisetin could reduce ROS [53]. Therefore, we believe that fisetin treatment may also reduce ROS in BM-MSCs.

These promising results support the in vivo testing of losartan and fisetin, either alone or in combination, to improve age-related fracture repair. We hypothesize that the multimodal positive effect on bone regeneration would be achieved by promoting osteogenesis and simultaneously reducing cellular senescence, inflammation/SASP, TGFβ, and osteoclast formation. We can further envision therapeutic applications in which losartan and/or fisetin are supplied in a sustained release for tissue engineering applications, such as our prior study where we developed sustained-release losartan nanofibers for maintaining a chondrogenic phenotype [52].

## Figures and Tables

**Table 1:** qPCR primer sequences.

Gene	Orientation	Sequence (5′–3′)
GAPDH	Forward	GGAGCGAGATCCCTCCAAAAT
	Reverse	GGCTGTTGTCATACTTCTCATGG

IL-1β	Forward	CCTGGACTTTCCTGTTGTCTAC
	Reverse	AAGTGAGTAGGAGAGGTGAGAG

TGF-β	Forward	GCTCGATCCTCTGCTCATTC
	Reverse	GGTTTTCCGCTTCAATGTGT

FABP4	Forward	ACTGGGCCAGGAATTTGACG
	Reverse	CTCGTGGAAGTGACGCCTT

PPARγ	Forward	TACTGTCGGTTTCAGAAATGCC
	Reverse	GTCAGCGGACTCTGGATTCAG

CEBP	Forward	ACTCCAGGGGTGAACGGAAT
	Reverse	CATGGGCGAACTCTTTTTGCT

COL1A	Forward	AGGGCTCCAACGAGATCGAGATCCG
	Reverse	TACAGGAAGCAGACAGGGCCAACGTCG

OC	Forward	GGCGCTACCTGTATCAATGG
	Reverse	GTGGTCAGCCAACTCGTCA

ALP	Forward	GACCTCCTCGGAAGACACTC
	Reverse	TGAAGGGCTTCTTGTCTGTG

## Data Availability

The data underlying this article will be shared on reasonable request to the corresponding author.
